# Predictors of Blood Culture Ordering and Results for Febrile Infants Aged 91 to 365 Days in the Emergency Department

**DOI:** 10.1016/j.acepjo.2026.100515

**Published:** 2026-10-21

**Authors:** Cole J. Florio, Madeleine M. Hatch, Madeline J. Somers, Tara L. Greenhow, Mary E. Reed, Dustin W. Ballard, David R. Vinson, Scott D. Casey

**Affiliations:** 1 California Northstate University College of Medicine, Elk Grove, California, USA; 2 Kaiser Permanente CREST Network, Pleasanton, California, USA; 3 Department of Emergency Medicine, UC Davis Health, Sacramento, California, USA; 4 Kaiser Permanente Northern California Division of Research, Pleasanton, California, USA; 5 Department of Pediatric Infectious Disease, Kaiser Permanente San Francisco, San Francisco, California, USA; 6 Department of Emergency Medicine, Kaiser Permanente San Rafael Medical Center, San Rafael, California, USA; 7 Department of Emergency Medicine, Kaiser Permanente Roseville Medical Center, Roseville, California, USA; 8 Department of Emergency Medicine, Kaiser Permanente Vallejo Medical Center, Vallejo, California, USA

**Keywords:** bacteremia, blood cultures, emergency department, fever, infant

## Abstract

**Introduction:**

The risk of invasive bacterial infections has dropped significantly among febrile infants due to widespread vaccination efforts. In older infants (age 91 days and older), diagnostic guidance is limited, and blood culture ordering is based on clinical judgment. We sought to evaluate blood culture ordering practice, bacteremia incidence, and the patient characteristics of true positive blood culture results.

**Methods:**

We conducted a retrospective cohort study of febrile pediatric patients aged 91 to 365 days who presented to Kaiser Permanente Northern California emergency departments (EDs) between January 1, 2018, and December 31, 2019, who had blood cultures drawn. We identified variables related to blood culture ordering using a generalized linear mixed model and adjudicated all positive results to determine rates of true bacteremia or contamination, as well as clinical characteristics of children with true positive bacteremia.

**Results:**

Among 5543 febrile infants, 611 (11.0%) underwent blood culture testing. After adjustment for covariates, higher ED temperature (adjusted odds ratio [aOR] 1.40, 95% CI, 1.30 to 1.50), presence of comorbidities (aOR 2.55, 95% CI, 2.00 to 3.25), and younger age (aOR 0.996, 95% CI, 0.995 to 0.998, *P* < .001) were associated with blood culture ordering. Of the 37 (6.1%) positive blood cultures, only 16 (2.6%) were true positives. All true positives occurred in infants with either an identifiable risk factor or a clinically evident source of infection. There were no cases of occult bacteremia identified.

**Conclusion:**

In febrile infants aged 91 to 365 days, true bacteremia was rare and associated with significant comorbidities or an obvious infectious source. Our findings suggest a more directed approach to ED blood culture ordering.

**The Bottom Line**

Blood cultures are commonly ordered for well-appearing, febrile children without bacteremia risk factors in the emergency department and false-positive results have consequences for patients. For children aged <60 days, several evidence-based algorithms exist to guide physician blood culture ordering practices, although there is minimal guidance for older children. This study examined variables associated with a physician’s decision to order blood cultures for febrile children aged 91 to 365 days, blood culture results, and incidence of occult bacteremia (bacteremia in a well-appearing febrile [temperature ≥39 °C/102.2 °F] child lacking risk factors or an identifiable source). True bacteremia was rare and associated with significant comorbidities or an obvious infectious source, and no cases of occult bacteremia were identified. The study indicates that bacteremia is rare in this age group and patients may benefit from a more judicious approach to blood culture ordering.

## 1 Introduction

### 1.1 Background

Fever accounts for approximately 15% to 21% of all pediatric emergency department (ED) visits.^1^^,^^2^ Although most pediatric fevers are attributable to benign, self-limited viral infections, invasive bacterial infections (IBIs), such as meningitis and bacteremia, must be considered.^1^^,^^3^^,^^4^ Occult bacteremia is defined as bacteremia in a well-appearing febrile child (temperature ≥39 °C/102.2 °F) without an identifiable source.^5^ In the prevaccine era, occult bacteremia was observed in 2% to 5% of well-appearing febrile children aged 2 to 36 months, prompting frequent blood culture ordering and antibiotic use.^5^, ^6^, ^7^ The introduction of the Hib vaccine in 1985 and the 7-valent pneumococcal conjugate vaccine (PCV7) in 2000 led to substantial declines in bacteremia and occult bacteremia incidence.^8^ The subsequent introduction of the 13-valent pneumococcal conjugate vaccine (PCV13) in 2010 further reduced the incidence by half.^9^^,^^10^

### 1.2 Importance

The decision to obtain a blood culture can be consequential, as false-positive results can cascade into unnecessary antibiotic treatments, prolonged hospitalizations, and additional invasive testing.^11^, ^12^, ^13^ These downstream effects not only increase health care costs but also impose stress on patients and families. For younger (aged <60 days) febrile children, evidence-based algorithms are available to guide physicians in blood culture ordering. However, the decision to obtain blood cultures for older children is guided by clinical gestalt. Given the marked reduction of pediatric bacteremia, routine blood culture ordering now merits a critical reevaluation in the context of modern, evidence-based practice.

### 1.3 Goals

The goal of this study was to determine: (1) physician blood culture ordering practice; (2) bacteremia and occult bacteremia incidence; and (3) patient characteristics of true positive blood culture results. We sought to describe blood culture results in our EDs, including rates of true positive and false positive rates. We predicted frequent blood culture ordering despite a low bacteremia incidence in our cohort, all of whom had an assigned pediatrician and access to routine childhood vaccines. We further hypothesized that there would be patient-level and clinical-level characteristics that would predict blood culture ordering in the ED. We anticipated a low incidence of occult bacteremia.

## 2 Methods

### 2.1 Design and Setting

We conducted a retrospective cohort study using electronic health record (EHR) data from febrile pediatric patients presenting to a community ED within Kaiser Permanente Northern California (KPNC). KPNC is an integrated health care system that is demographically and socioeconomically diverse and broadly representative of the surrounding region.^14^ This study was approved by the KPNC Institutional Review Board with a waiver of informed consent. All participating EDs had continuous access to onsite diagnostic testing, including routine laboratory services. Treating physicians were residency-trained and board-certified or board-eligible emergency medicine physicians. A small minority were fellowship-trained in pediatric emergency medicine. Pediatric hospital medicine physicians were available for consultation 24 hours per day, 7 days per week. Facility-level point-of-care influenza and respiratory syncytial virus (RSV) testing was available seasonally during winter months**,** whereas comprehensive respiratory viral panels (including influenza, RSV, human metapneumovirus, rhinovirus, and parainfluenza) were available year-round as send-out tests processed by the microbiology laboratory. The decision to obtain respiratory viral testing and blood cultures, as well as the timing of these tests, was at the discretion of the treating emergency physician. Data were collected from January 1, 2018, to December 31, 2019, prior to the onset of the COVID-19 pandemic.

### 2.2 Study Population

We identified all KPNC health plan members aged 91 to 365 days who presented to the ED with a documented fever. Fever was defined as a temperature ≥100.4 °F (38.0 °C) at triage or within 4 hours of arrival in the ED, or a documented fever at another healthcare encounter within 12 hours preceding the ED visit (Fig 1). Patients who did not meet the documentation criteria for fever or who had incomplete data were excluded.

**Figure 1:**
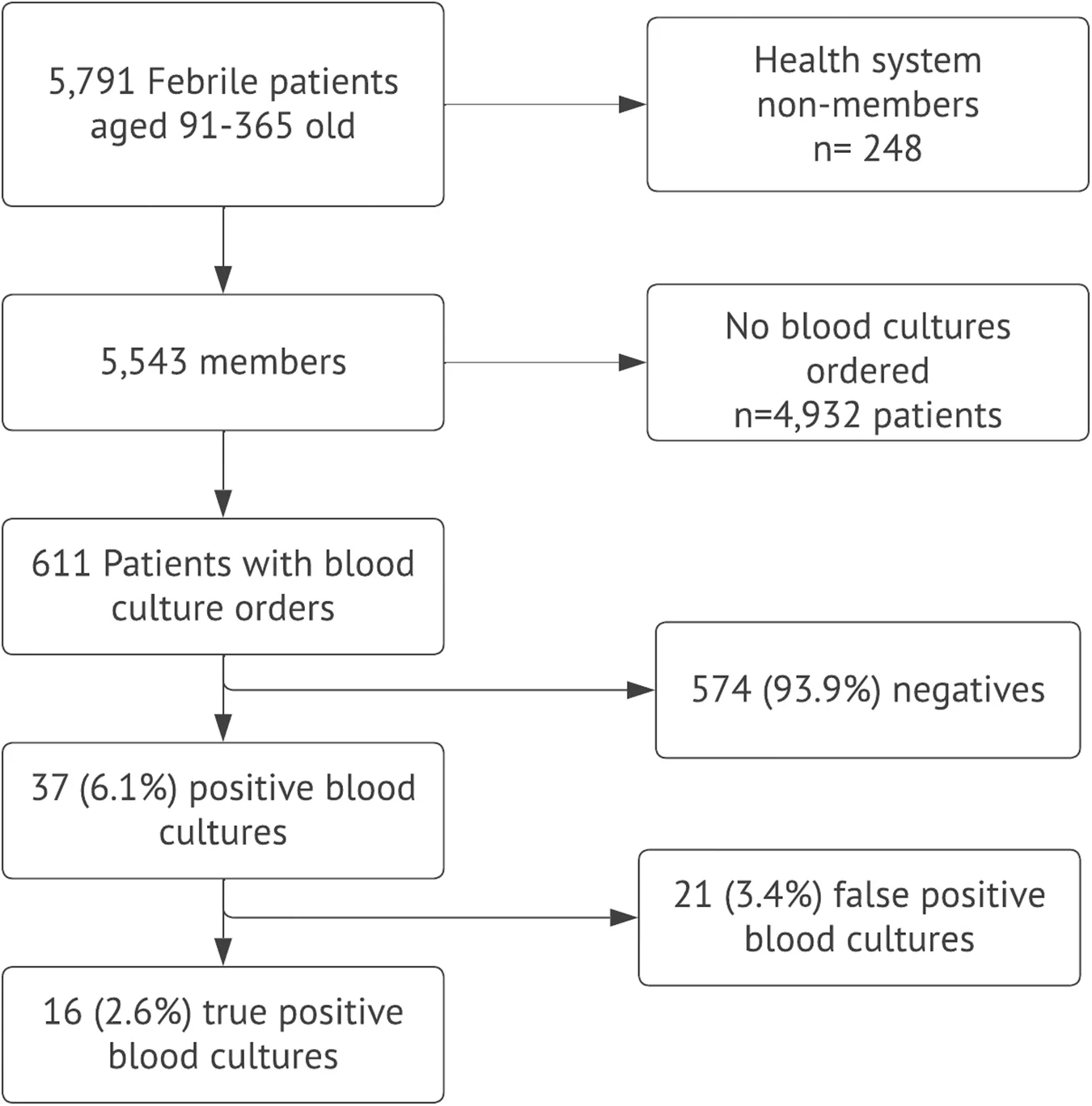
Cohort assembly.

### 2.3 Data Collection, Definitions, and Outcomes

Abstractors performed manual chart review after completing structured training on data collection methods using a standardized computerized data collection tool. The principal investigator answered abstraction questions throughout the study. Race and ethnicity data were self-reported by families. Prematurity was defined based on the presence of ICD-10 diagnostic codes for preterm birth documented in the electronic health record. Underimmunization was defined based on the presence of ICD-10 code (Z28.39). Comorbidities were defined using the pediatric complex chronic conditions classification system developed by Feudtner et al., which defines these conditions as medical conditions expected to last at least 12 months (unless death intervenes) and to involve either multiple organ systems or one organ system severely enough to require specialty pediatric care and likely hospitalization.^15^

Respiratory viral status was categorized as positive for influenza A or B, RSV, both influenza and RSV, or negative. The primary outcome was blood culture ordering and identification of patient characteristics associated with blood culture utilization. The secondary outcomes were the results of the blood culture (positive or negative) and the patient characteristics and diagnoses associated with positive results. All positive blood cultures were reviewed by a pediatric infectious disease physician (T.L.G.) and classified as either a true or false positive (contaminant). This determination was based upon the clinical history, microbiologic findings, laboratory results, respiratory viral testing (when available), the presence of an identifiable infectious source, and the patient's clinical course. Organisms classified as contaminants included coagulase-negative staphylococci, *Streptococcus* *mitis*, *Lactococcus* species, *Bacillus* species (excluding Bacillus anthracis), Corynebacterium species, Micrococcus species, Sphingomonas paucimobilis, and Staphylococcus hominis.

### 2.4 Statistical Analysis

We used descriptive statistics to summarize patient characteristics, reporting categorical variables as counts and proportions, and continuous variables as medians with interquartile ranges (IQRs). Bivariate comparisons between patients who did and did not have blood cultures ordered were made using the Wilcoxon rank-sum test, Pearson’s chi-square, or Fisher’s exact test, as indicated. To identify characteristics associated with blood culture ordering, we constructed a generalized linear mixed model, adjusting for demographic and clinical covariates with a random intercept to capture within-subject correlation (due to a small number of repeat ED visits). Results are reported as adjusted odds ratios (aORs) with 95% CIs. A *P* value of <.05 was considered statistically significant. All statistical analyses were conducted using R version 4.3.1 and SAS version 9.4.

## 3 Results

We identified 5543 febrile infants aged 91 to 365 days who presented to one of KPNC’s community EDs between 2018 and 2019 (Fig 1). Of these, 611 (11%) underwent blood culture testing. Among the 611 patients who had blood cultures ordered, the median age was 240 days with a median maximum fever of 102.4 °F (Table 1). Compared with patients who did not have cultures ordered, patients with blood cultures ordered were younger (240.0 days vs 260.0 days, *P* < .001), had a higher maximum ED temperature (102.4 °F vs 102.0 °F, *P* < .001), and more frequently had a comorbidity (21% vs 9.6%, *P* < .001).

**Table 1:** Characteristics of febrile infants aged 91 to 365 days by blood culture ordering status.

Characteristic	Overall	Blood culture ordered?		
	n = 5543^1^	No, n = 4932^1^	Yes, n = 611^1^	*P* value
Age (days)	259.00 (198.5-319.5)	260.00 (201.0-319.0)	240.00 (163.3-316.8)	<.001
Sex				>.9
Female	2532 (46)	2252 (46)	280 (46)	
Male	3011 (54)	2680 (54)	331 (54)	
Race/ethnicity				.16
Asian	1039 (19)	910 (18)	129 (21)	
Black	501 (9.0)	460 (9.3)	41 (6.7)	
Hispanic	1985 (36)	1765 (36)	220 (36)	
Other^2^	657 (12)	589 (12)	68 (11)	
Non-Hispanic White	1361 (25)	1208 (24)	153 (25)	
Max ED temperature °F	102.0 (101.1-103.0)	102.0 (101.1-103.0)	102.4 (101.4-103.4)	<.001
Comorbidity	601 (11)	472 (9.6)	129 (21)	<.001
Prematurity	523 (9.4)	449 (9.1)	74 (12)	.016
Influenza A or B only	219 (4.0)	188 (3.8)	31 (5.1)	.13
RSV	485 (8.7)	423 (8.6)	62 (10)	.20
Underimmunized	680 (12)	595 (12)	85 (14)	.20

1 n (%) throughout, except age and max ED temperature, which are reported as continuous variables with medians (IQR).

2 Native Hawaiian or Pacific Islander, American Indian/Alaskan Native, Decline to state.

After adjustment, higher ED temperature (aOR 1.40, 95% CI, 1.30 to 1.50), presence of comorbidities (aOR 2.55, 95% CI, 2.00 to 3.25), and younger age (aOR 0.996, 95% CI, 0.995 to 0.998, *P* < .001) were independently associated with blood culture ordering (Table 2). Underimmunization, however, was not significantly associated with blood culture ordering (aOR 1.28, 95% CI, 0.98 to 1.67, *P* = .065).

**Table 2:** Association of variables with blood culture ordering.

Characteristic	Adjusted OR	95% CI	*P* value
Age (days)	0.996	0.995, 0.998	<.001
Sex			.93
Female	—	—	
Male	0.99	0.83, 1.19	
Race/ethnicity			.12
Asian	—	—	
Black	0.61	0.41, 0.91	
Hispanic	0.88	0.69, 1.14	
Other^1^	0.89	0.64, 1.23	
White	1.00	0.77, 1.32	
Max ED temperature °F	1.40	1.30, 1.50	<.001
Comorbidity	2.55	2.00, 3.25	<.001
Prematurity	1.19	0.90, 1.59	.22
Influenza A or B only			.17
Influenza A or B	—	—	
Other	0.76	0.51, 1.13	
RSV	1.07	0.78, 1.48	.67
Underimmunized	1.28	0.98, 1.67	.065

OR, odds ratio; ED, emergency department.

1 Native Hawaiian or Pacific Islander, American Indian/Alaskan Native, Decline to state.

Among the 611 patients with blood cultures, 574 (93.9%) were negative, and 37 (6.1%) were positive (Fig 2). Of these positive cultures, 21 (3.4%) were false positives, and 16 (2.6%) were true positives for bacteremia (positive predictive value of 43%) (see Appendix for more details on true positives). Among patients with true positive cultures, 6 (37.5%) had identifiable risk factors, such as indwelling catheters, congenital cardiac defects, genitourinary abnormalities, or prolonged fever. The remaining 10 (62.5%) had a clinically evident infection, including urinary tract infection, otitis media, diarrhea, cellulitis, or meningitis. Among patients who underwent blood culture testing, there were no identified cases of occult bacteremia; no patient without an identifiable infectious source had a true positive blood culture. Two patients had bacteremia and meningitis without an apparent source: the first patient presented with 10 days of fever and the second had a recent hospitalization for suspected intussusception who was discharged after a normal contrast enema but was called back to the ED after both blood cultures grew *Streptococcus pneumoniae*.

**Figure 2:**
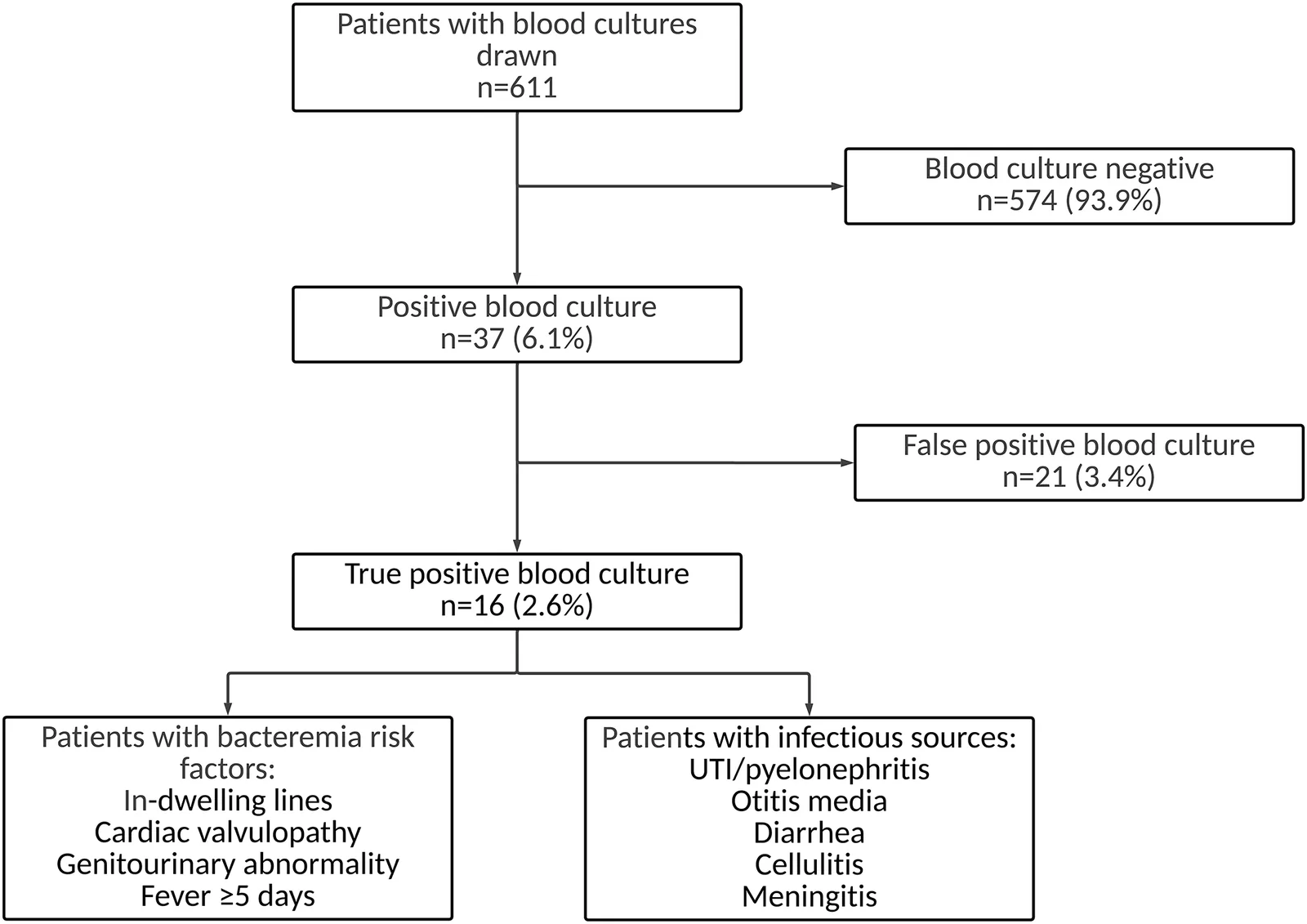
Blood culture results. GU, genitourinal; UTI, urinary tract infection.

## 4 Limitations

Our study has several limitations. First, as a retrospective analysis, our findings are subject to limitations inherent to EHR data, including potential misclassification of comorbidities, fever degree, or culture results. Additionally, we were unable to assess several factors which are not routinely captured in the EHR that may influence blood culture ordering decisions, including patient appearance, parental concern, clinician judgment, perceived illness severity, and other contextual features of the clinical encounter. Consequently, residual confounding by these unmeasured factors is possible. Second, our study is subject to potential selection bias because blood cultures were obtained at the discretion of the treating clinician rather than systematically in all febrile infants. As a result, children perceived to be at lower risk for bacteremia may not have undergone blood culture testing, and we cannot exclude the possibility that occult bacteremia occurred among untested patients. However, this approach reflects contemporary clinical practice, in which blood cultures are obtained selectively based on physician assessment rather than routinely in all febrile infants. Third, our blood culture sample size was modest, which limited power to evaluate less common subgroups, such as specific comorbidity types or incomplete immunization status. Fourth, although our cohort was drawn from a large integrated healthcare system that is broadly representative of the surrounding Northern California population, practice patterns, healthcare access, and contamination rates may differ in other settings, limiting generalizability. Each member in our health system is assigned a pediatrician, which may affect downstream development of IBI and bacteremia. A higher vaccination rate might also be assumed in a cohort, such as ours. Finally, the study evaluated patients from 2018 through 2019. Changes in blood culture ordering practices, pathogen epidemiology, and antimicrobial resistance patterns since that study period may limit the applicability of these findings to current clinical practice.

## 5 Discussion

In this community-based cohort, we examined blood culture ordering and results, blood culture test characteristics for bacteremia detection, and factors associated with pediatric blood culture ordering and results in infants 91 to 365 days of age. Evidence-based algorithms, such as Roseville, American Academy of Pediatrics, and the Pediatric Emergency Care Applied Research Network criteria, aid in identifying febrile infants at risk for invasive bacterial infection in the ED.^16^, ^17^, ^18^, ^19^ Given the lower risk for IBIs in older infants, physicians currently lack guidance on blood culture ordering for children older than 90 days.^20^^,^^21^

Bacteremia was uncommon in our cohort (<0.3%), compared with 0.9% reported in previous studies of infants aged 57 to 180 days.^22^ We noted a high proportion of false-positive results (3.4%), which was roughly double the rate noted previously in a similar population in our health system, although the cited study included the ED and other sites of care.^23^ Prior research has shown that blood cultures obtained in the ED have the highest contamination rates across hospital settings.^9^ The number of contaminated blood cultures we report is similar to previous reports.^24^, ^25^, ^26^, ^27^, ^28^ Contaminated blood cultures increase the risk of invasive testing, unnecessary antibiotic exposure, and undue stress on patients and their families. Phlebotomist teams, diversion devices, and blood culture collection training of ED staff may decrease the false-positive rate as recommended by the American Society for Microbiology.^29^^,^^30^

The presence of comorbidities (congenital heart disease, genitourinary anomalies, indwelling catheters) was associated with greater likelihood of blood culture ordering. Physicians likely correctly attribute higher risk to this population.^31^ Higher ED temperatures were associated with increased blood culture ordering. Prior studies have shown that higher temperatures may be predictive of IBIs in younger infants, although the risk is unclear in those >3 months of age. Interestingly, an association between immunization status and blood culture ordering was not observed. The relevance of nonimmunized or underimmunized status, as an independent risk factor for bacteremia, has decreased due to herd immunity. Physicians may also be unaware of a child’s immunization status during the ED encounter. Prior studies have found that underimmunized children may carry a slightly elevated relative risk for IBI; however, the absolute risk remains low.^9^

In conclusion, true bacteremia was uncommon among febrile infants aged 91–365 days who underwent blood culture testing in our community emergency departments. Blood cultures were most frequently obtained in younger infants, those with higher temperatures, and those with significant comorbidities. Among tested patients, occult bacteremia was not identified. The patient with a positive blood culture whose risk factor was fever >5 days presented with 10 days of fever. The infant was diagnosed with meningitis by CSF culture, though the source was not obvious by physical examination alone. The second patient presented on the fifth day of fever after a recent hospitalization and they were hospitalized for bacteremia and meningitis. These findings provide insight into blood culture ordering practices and diagnostic yield in a post-PCV-era population, although prospective studies that systematically evaluate both tested and untested patients are needed before recommendations regarding blood culture utilization can be made.

## Author Contributions

C.J.F., M.M.H., D.R.V., and S.D.C. conceived and designed the study. S.D.C. and D.R.V. supervised the study. M.J.S. performed the statistical analyses. M.J.S., M.E.R., D.W.B., T.L.G., and S.D.C. conducted data abstraction. T.L.G. adjudicated blood culture results and provided infectious disease expertise. C.J.F., M.M.H., D.R.V., and S.D.C. interpreted the data. C.J.F., M.M.H., D.R.V., and S.D.C. drafted the manuscript, and all authors contributed substantially to its revision. S.D.C. takes responsibility for the paper as a whole.

## Funding and Support

By *JACEP Open* policy, all authors are required to disclose any and all commercial, financial, and other relationships in any way related to the subject of this article as per ICMJE conflict of interest guidelines (see www.icmje.org). This study was funded by the Garfield Memorial Fund.

## Conflict of Interest

All authors have affirmed they have no conflicts of interest to declare.

## Data Sharing Statement

The data that support the findings of this study are not publicly available due to institutional restrictions and protections on patient privacy.
