# Supplementary material for: Predictors of Blood Culture Ordering and Results for Febrile Infants Aged 91 to 365 Days in the Emergency Department
Source: J Am Coll Emerg Physicians Open. 2026 Oct 21;7(6):100515. doi: 10.1016/j.acepjo.2026.100515 (PMC13717012; doi:10.1016/j.acepjo.2026.100515)
Supplement: Supplementary file 1 [file mmc1.docx]

**Appendix 1:** Patient and laboratory characteristics of patients with adjudicated true positive blood cultures.

| **Organism** | **CSF** | **Meningitis** | **CSF culture** | **Source** | **Fever duration (days)** | **Note** |
| --- | --- | --- | --- | --- | --- | --- |
| Haemophilus influenzae | Pleocytosis | Yes | Haemophilus influenzae | Otitis media | 4 |  |
| Enterobacter cloacae Complex |  | No |  | Unknown, E coli UTI | 11 |  |
| Streptococcus pyogenes (Group A) |  | No |  | Port infection | 1 |  |
| Escherichia coli | Pleocytosis | No |  | UTI/pyelonephritis | 1 | febrile seizure |
| Escherichia coli |  | No |  | UTI | 2 |  |
| Escherichia coli |  | No |  | UTI | 1 | Known severe VUR |
| Staphylococcus aureus |  | No |  | line infection | 1 | Short gut |
| Staphylococcus aureus |  | No |  | septic arthropathy | 3 |  |
| Escherichia coli |  | No |  | UTI | 1 |  |
| Salmonella species |  | No |  | diarrhea | 3 | Recent international travel, stool culture growing Salmonella |
| Escherichia coli |  | No |  | UTI | 1 |  |
| Streptococcus pyogenes (Group A) |  | No |  | cellulitis | 2 |  |
| Klebsiella oxytoca | Klebsiella oxytoca | Yes | Klebsiella oxytoca | VP shunt infection | 1 | Ex-premature with history of E coli meningitis |
| Staphylococcus aureus |  | No |  | Likely cardiac | 1 | Williams syndrome, presented after cardiac surgery |
| Streptococcus pneumoniae | Abnormal CSF | Probable |  | UTI | 1 | treated for meningitis, culture obtained after antibiotics |
| Haemophilus influenzae serotype A,C,D,E or F | Haemophilus influenzae | Yes | Haemophilus influenzae |  | 10 |  |

Abbreviations: UTI, urinary tract infection; VUR, vesicoureteral reflux; VP, ventriculoperitoneal.
